# Hematological Reference Values for Healthy Adults in Togo

**DOI:** 10.5402/2011/736062

**Published:** 2010-11-07

**Authors:** Irenee Messanh Kueviakoe, Akuete Yvon Segbena, Helene Jouault, Ahoefa Vovor, Michele Imbert

**Affiliations:** ^1^Hematology Laboratory, Campus Teaching Hospital, University of Lome, 03 bp 30284 Lome-Togo, Togo; ^2^Hematology Laboratory, Henri Mondor Hospital, 94000 Creteil, France; ^3^Hematology Laboratory, Tokoin Teaching Hospital, Lome, 01 bp57 Lome-Togo, Togo

## Abstract

The hematological reference values are very important for diagnostic orientation and treatment decision. The aim of this study was to establish hematological reference values for healthy adults in Togo. A total of 2571 voluntary blood donors participated to this study. Only 1349 subjects negative for HIV, HBV, HCV, malaria, and without hemoglobin abnormalities in electrophoresis and hypochromia on blood smear, were definitively retained for the study. Median hemoglobin level was higher in males than females (15.1 g/dL versus 13.0 g/dL, *p* = 0.000). Median total WBC (4.2×10^9^/L) and absolute neutrophil counts (1.6×10^9^/L) were similar by gender. The median lymphocyte counts in males and females were, respectively, 2.1×10^9^/L and 2.2×10^9^/L (*p* = 0.11). The median platelet count was lower in males than females (236×10^9^/L versus 247×10^9^/L, *p* = 0.004). Our median values for RBC parameters differ from those of African countries probably because of our inclusion criteria which eliminate most cases with iron deficiency and/or thalassemia.

## 1. Introduction

In daily practice in Togo, it is important to have hematological reference values for diagnosis orientation and treatment decision. The hematological reference values were determined many years ago for the Caucasian's populations [1–3]. Recently, several authors tried to establish reference values in hematology for African countries. However there are some discrepancies from one study to another which may be related to different factors such as age, sex, geographic origin, altitude, and ethnic origin [4–10]. Moreover, many constitutional hemoglobin abnormalities (thalassemia, sickle cell disease and hemoglobin C) or pathologic conditions (malaria, HIV, HBV and HCV viral infections), influence the hematological values.

Togo is a Western African country, with an altitude below 1000 meters. Until now, hematological reference values have not been established. The values usually used are those of Caucasian populations. In Togo, it is especially difficult to establish hematological reference values for RBC parameters because of the (i) high frequency of alpha-thalassemia (49.2%), (ii) beta-globin gene abnormalities (37.5%), [11], and, (iii) iron deficiency anemia in the overall population. 

According to this context and for a better interpretation of hematologic results, we decided to establish hematological reference values in Togo by studying healthy Togolese adults from voluntary blood donors recruited in the National Blood Transfusion Center of Lomé.

## 2. Subjects and Methods

### 2.1. Subjects

Between April 2008 and September 2008, 2571 blood donor volunteers (less than three donations) were received at the National Blood Transfusion Center of Lomé and gave their informed consent for the study. For each of them, a total blood cell count was performed. Moreover, a peripheral blood film was done in order to detect abnormalities of RBC (mainly hypochromia) and all samples were tested for HIV, HBV, and HCV viral infection, and malaria. The classical method of hemoglobin electrophoresis with integration by densitometry was performed for thalassemia screening in all cases. 

The criteria for inclusion in our study were the following: negativity of all serological tests for viral infections and for malaria, normal hemoglobin electrophoresis screening, and absence of hypochromia on peripheral blood smear. According to these criteria, only 1349 (52%) subjects were finally retained for the study.

### 2.2. Blood Collection

All samples were collected between 7.00 a.m. and 11.30 a.m. at the National Blood Transfusion Center of Lomé and analyzed at the day of collection.

All blood was collected with a Vacutainer system in 5 mL EDTA tubes. The serology tests for HIV, HBV, and HCV detections were performed by ELISA (enzyme-linked immunosorbent assay) in the National Blood Transfusion Center of Lomé. Peripheral blood film was performed and stained according to MGG technique. Malaria was tested with the thick drop method.

### 2.3. Hematological Analysis

A Sysmex SF-3000 automated hematology analyzer was used for whole-blood analysis of hematological parameters. 

The instrument automatically counts and gives a printout result of absolute numbers of leukocytes (WBC) (10^9^/L), erythrocytes (RBC) (10^12^/L), hemoglobin (g/dL), hematocrit (%), MCV (fl), MCH (pg), MCHC (g/dL), platelets (10^9^/L), neutrophils (10^9^/L), lymphocytes (10^9^/L), eosinophils (10^9^/L), basophils (10^9^/L), and monocytic cells (10^9^/L).

### 2.4. Statistical Analysis

Data was recorded in the Excel software and analyzed with Epi Info 3.3.2 software. The mean, median, and standard deviation values were calculated for each parameter. Kruskal-Wallis test for two groups was used to compare parameters according to gender. Differences were statistically significant when *p* < 0.05.

## 3. Results

Among the 1379 blood donor volunteers, 1047 were males (77.6%) and 302 were females (22.4%); their age ranged from 17 to 58 years. 

The means, medians, and 95th percentile references values according to gender for RBC parameters (RBC count, hemoglobin, hematocrit, and hematimetric constants) are presented in Table 1.

Males had higher median RBC (5.0 × 10^12^/L versus 4.5 × 10^12^/L), hemoglobin (15.1 g/dL versus 13.0 g/dL), hematocrit (42.8% versus 38.1%), MCV (85 fl versus 84 fl), and MCH (29.7 pg versus 29.3 pg) than females. The difference of RBC, hemoglobin, hematocrit, MCV, and MCH by gender was statistically different (*p* < 0.05). The medians of MCHC were 35.1 g/dL in males and females. No gender difference was observed for MCHC (*p* = 0.08). 

The Table 2 shows the means, medians, and 95th percentile of WBC parameters (WBC, neutrophils, eosinophils, lymphocytes and monocytes counts). The ranges of total WBC count, absolute neutrophil count, and absolute lymphocyte count were, respectively, 1.9–10.1 × 10^9^/L, 0.5–5.4 × 10^9^/L and 1.1–4.3 × 10^9^/L. No gender difference was noticed for WBC parameters (*p* > 0.11). 

The median platelet count in males was 236 × 10^9^/L (range: 120–443 × 10^9^/L) and 247 × 10^9^/L (range: 150–436 × 10^9^/L) in females. The means by gender were, respectively, 249 ± 61.2 × 10^9^/L for males and 249 ± 61.2 × 10^9^/L for females. Males had lower platelet count than females and the difference was statistically significant (*p* = 0.004).

## 4. Discussion

The aim of our study was to establish hematological reference values which may serve as western African standards for interpretation of laboratory results. Many factors influence the hematological values such as sex, age, ethnic origin, geographic location, season, and genetic disease [1, 2, 13]. Moreover, one of the main problems in African countries is to determined “normal healthy individuals” due to the high prevalence of hemoglobin abnormalities, iron deficiency, and viral and parasitic infections. Therefore, to date, all hematological reference values published for African population do not specify hemoglobin characteristics and iron status of the tested subjects.

The RBC parameters are influenced by several factors especially the abnormalities of hemoglobin synthesis. Those abnormalities can be constitutional (sickle cell disease, alpha- and beta-thalassemia) or acquired (iron deficiency). Thalassemia and iron deficiency display hypochromia. An ideal approach would be to realize high performance liquid chromatography (HPLC) and ferritin measurement to eliminate thalassemia and iron deficiency before selecting subjects for an RBC study. Until now, no publications for hematological reference values have tested all those parameters. In our study, we try to eliminate most of these abnormalities by performing standard hemoglobin electrophoresis and detecting hypochromia on the peripheral blood smear.

Our results as well as those of other African countries are displayed in Table 3. Medians of hemoglobin and hematocrit were higher in males than females, and differences were statistically significant. Our results are similar to those of the studies performed in African countries with an altitude below 2000 meters [5, 7, 9]. At a higher altitude (Ethiopia), results were different with higher hemoglobin and hematocrit levels [10, 12, 14]. In Kenyan's population [8], hemoglobin median was under 10 g/dL; this result was lower than in most of the other African population [4, 5, 7, 9, 15]. However, the authors did not specify the method used to establish reference sample collection. MCV median and ranges were, respectively, 87 fl and 68.8–97.7 fl. It can be hypothesized that the lower median hemoglobin and the low minimal range MCV, which contrast with the normal or higher RBC, may be linked to the presence of alpha-thalassemia. This study illustrates the difficulty to get healthy individuals for reference values for RBC series.

WBC differential values were obtained by an automated analyzer as reported in several other recent studies. However some authors still used a manual WBC differential count [7, 10]. The median WBC count was 4.1 × 10^9^/L with a range of 1.9–10.1 × 10^9^/L. Our limit values were lower than those found elsewhere, but the median values were similar to the results of other African studies [4, 5, 7, 8] except the median of WBC count found in the South African's study which was higher [12]. No difference by gender was found, except in Kenya where males had lower values than females. Neutropenia was found in all black African or American studies [5, 7, 10, 16]. The total WBC and absolute neutrophil count were significantly lower in the black population than in all of other groups [17, 18]. Mechanisms by which black population have lower values of neutrophils is still not clear. The hypothesis of an excess of marginated neutrophils pool is often proposed but a recent study did not confirm it [19].

The platelet count is globally lower among black people than in Caucasians. In our study, males had lower platelet count than females with a significant difference. In males, the median platelet count was 236 × 10^9^/L and in females 247 × 10^9^/L. The same results were observed in other African [4, 5, 7, 8, 20], European [1], and North American [1, 2] studies. Some authors accused malaria disease, but identical results were found in countries where malaria disease is not endemic (Ethiopia, Europe, North America). The reasons for these differences according to gender are still unclear.

## 5. Conclusion

In spite of the factors influencing hematological values, this study permitted to establish the hematological reference values in Togo. The median values are similar to the ones found in other studies performed in Africa and even in northern countries. The ranges of different parameters are often different especially for RBC considering the influence of alpha-thalassemia and iron deficiency. The reason for lower absolute neutrophil count is still unclear. In the absence of previously hematological reference values in Togo, we offered to use these results for clinical management of Togolese patients and interpretations of laboratory data for research purpose.

## Figures and Tables

**Table 1:** Means, medians, and 95th percentile references values by gender for RBC count, hemoglobin, hematocrit, and hematimetric constants.

Subject group (*n*) and parameters	RBC (10^12^/L)	Hemoglobin (g/dL)	Hematocrit (%)	MCV (fl)	MCH (pg)	MCHC (g/dL)
ALL (*n* = 1349)						
Means ± SD	4.8 ± 0.5	14.5 ± 1.7	41 ± 4.7	85 ± 4.1	30 ± 1.8	35 ± 1.4
Median	4.9	14.6	41.8	84.5	29.6	35.1
95% range	3.1–6.4	10–18.4	28–54	80–99	25–37	29–41

Male (*n* = 1047)						
Means ± SD	4.9 ± 0.5	14.8 ± 1.5	42 ± 4.2	86 ± 4.2	30 ± 1.8	35 ± 1.4
Median	5.0	15.1	42.8	85	29.7	35.1
95% range	3.3–6.4	10–18.4	28–54	80–99	26–36	29–39

Female (*n* = 302)						
Means ± SD	4.5 ± 0.5	13.1 ± 1.5	38 ± 4.4	84 ± 3.1	29 ± 1.6	35 ± 1.5
Median	4.5 (0.000)*	13 (0.000)*	38.1 (0.000)*	84 (0.000)*	29.3 (0.000)*	35.1 (0.08)**
95% range	3.1–6.0	10.3–17.1	28–47	80–95	25–37	30–41

*p  values < 0.05: statistically significant difference between males and females

**p  values > 0.05: no statistically significant difference between males and females.

**Table 2:** Means, medians, and 95th percentile references values by gender for WBC parameters.

Subject group (*n*) and parameters	WBC (10^9^/L)	Neutrophils (10^9^/L)	Eosinophils (10^9^/L)	Lymphocytes (10^9^/L)	Monocytes (10^9^/L)
ALL (*n* = 1349)					
Means ± SD	4.3 ± 1.1	1.7 ± 0.7	0.2 ± 0.07	2.2 ± 0.5	0.2 ± 0.1
Median	4.1	1.6	0.2	2.1	0.2
95% range	1.9–10.1	0.5–5.4	0–0.5	1.1–4.3	0.05–0.8

Male (*n* = 1047)					
Means ± SD	4.3 ± 1.0	1.7 ± 0.65	0.2 ± 0.05	2.2 ± 0.55	0.2 ± 0.13
Median	4.1	1.6	0.2	2.1	0.2
95% range	1.9–10.1	0.5–5.4	0–0.5	1.1–4.3	0.05–0.8

Female (*n* = 302)					
Means ± SD	4.4 ± 1.1	1.7 ± 0.71	0.2 ± 0.09	2.2 ± 0.57	0.2 ± 0.15
Median	4.2 (0.22)**	1.6 (0.78)**	0.2 (0.20)**	2.2 (0.11)**	0.2 (0.53)**
95% range	2.2–7.8	0.5–4.4	0–0.5	1.2–4.3	0.05–0.8

*p  values < 0.05: statistically significant difference between males and females

**p  values > 0.05: no statistically significant difference between males and females.

**Table 3:** Means, median, and 95th percentile for platelets count (10^9^/L) by gender.

	All	Males	Females
	(*n* = 1349)	(*n* = 1047)	(*n* = 302)
Means ± SD	249 ± 61.2	246 ± 61	257 ± 60
Median	239	236	247 (0.004)*
95th-percentile	120–443	120–443	150–436

*p  value < 0.05: statistically significant difference between males and females.

**Table 4:** Hematological reference values in African countries.

	RBC (10^12^/L)		Hemoglobin (g/dL)		Hematocrit (%)		MCV	WBC	Neutro	Lympho	Mono	Platelet
	Males	Females	Males	Females	Males	Females	(fl)	(10^9^/L)	(10^9^/L)	(10^9^/L)	(10^9^/L)	(10^9^/L)
Our study	3.3–6.4 (5.0)	3.1–6.0 (4.5)	10–18.4 (15.1)	10.3–17.1 (13)	28–54 (42.8)	28–47 (38.1)	80–99 (84.5)	1.9–10.1 (4.1)	0.5–5.4 (1.6)	1.1–4.3 (2.1)	0.05–0.8 (0.2)	120–443 (239)
Uganda [5]	3.8–6.0 (4.9)	3.7–5.3 (4.5)	11.1–16.8 (14.1)	10.1–14.3 (12.5)	32.2–47.8 (40.7)	29.6–41.4 (36.2)	67.7–95.2	3.4–8.7 (5.3)	0.84–3.37 (1.8)	1.4–4.2 (2.4)	0.17–0.59 (0.32)	100–297 (198)
Central African [7]	4.5–6.1 (5.14)	3.42–5.44 (4.5)	12.3–17.3 (14.9)	9.1–14.9 (12.5)	39–52 (45)	28–44 (38)	NA	2.9–8.3 (5.05)	NA	1.4–4.2 (2.5)	NA	117–382 (228)
Ethiopia [10]	4.3–5.9 (5.1)	3.7–5.2 (4.5)	13.9–18.3 (16.1)	12.2–16.6 (14.3)	41.6–55.1 (48.3)	35.3–48.8 (42)	NA	3.0–10.2 (6.1)	1.05–7.18 (2.77)	0.96–3.47 (1.8)	0.17–0.69 (0.32)	98–337 (205)
South Africa [12]	3.2–5.8 (4.6)	3.0–5.3 (4.2)	10.3–16.7 (14)	9.0–15.2 (12.4)	31–52.5 (42.3)	27.3–47.2 (37.6)	NA	3.7–12.6 (7.2)	NA	NA	NA	NA
Gambia [4]	NA	NA	11.1–16.6	9.8–15.0	NA	NA	84.3–87.5	3.3–8.4	NA	NA	NA	124–397
Kenya [8]	4.4–6.3 (5.3)	3.7–5.6 (4.8)	8.3–11.3 (9.9)	5.9–10 (8.44)	40–50 (47)	30–50 (40)	68.8–97.2 (87)	2.8–8.2 (4.4)	0.9–4.7 (1.85)	1.14–3.45 (1.95)	0.13–0.6 (0.29)	120–411 (226)
Tanzania [9]	4.4–6.3	3.8–5.6	13.7–17.7	11.1–15.7	40.2–53.7	36.2–46.8	NA	3.0–7.9	1.1–4.7	NA	NA	150–395

*(Reference ranges) and median values, NA: not available, Neutro: neutrophils, Lympho: lymphocytes, and Mono: monocytes.
